# Extracellular matrix remodeling and regulated cell-death-immune associations in adhesive capsulitis: a three-cohort transcriptomic analysis

**DOI:** 10.3389/fcell.2026.1951682

**Published:** 2026-10-16

**Authors:** Lin Huang, Ling Cheng, Jin He, Rui Zhang, Xia Li, Qiurong Xia

**Affiliations:** 1 Department of Integrated Traditional Chinese and Western Medicine and Rheumatology, Dianjiang People’s Hospital of Chongqing, Chongqing, China; 2 Department of Traditional Chinese Medicine, Yongxing Town Central Health Center, Chongqing, China

**Keywords:** adhesive capsulitis, extracellular matrix, ferroptosis, necroptosis, pyroptosis, transcriptomics

## Abstract

**Background:**

Adhesive capsulitis (AC) is a fibroinflammatory disorder characterized by painful shoulder restriction and progressive capsular fibrosis. Whether regulated cell-death (RCD) programs are consistently altered across independent transcriptomic cohorts remains uncertain.

**Methods:**

We reanalyzed three public bulk RNA-seq cohorts. GSE140731 was the discovery cohort (22 AC and 26 shoulder-instability surgical controls), and GSE190023 and GSE238053 were independent validation cohorts. Differential expression was estimated from filtered raw counts with PyDESeq2, with age/sex adjustment in GSE140731. Curated pyroptosis, ferroptosis, and necroptosis gene sets; immune marker–gene scores; pathway enrichment; STRING network structure; cross-cohort concordance; an exploratory three-gene classifier; and qPCR validation of four selected genes in TGF-β1-stimulated SW982 cells were evaluated.

**Results:**

The adjusted discovery analysis identified 439 upregulated and 113 downregulated genes at FDR < 0.05. ECM–receptor interaction was strongly positively enriched (NES = 2.299, q = 0.001), making extracellular-matrix remodeling the clearest pathway-level signal. RCD findings were mixed: pyroptosis was not different (delta = −0.105, q = 0.708), ferroptosis was lower but borderline after FDR correction (delta = −0.426, q = 0.056), and necroptosis was lower (delta = −0.320, q = 0.035); the core necroptosis gene set was also negatively enriched (NES = −1.673, q = 0.038). Six immune marker scores were FDR-significant in discovery, but none replicated at cohort-wise FDR significance in the validation cohorts. Cross-cohort gene-effect concordance was heterogeneous (Spearman rho = −0.047 for GSE190023 and 0.383 for GSE238053). The COL11A1–SPON2–PITPNC1 classifier achieved a pooled out-of-fold AUC of 0.820 (95% CI, 0.684–0.930) in GSE140731; external-cohort AUCs ranged from 0.750 to 0.812 with wide confidence intervals, and non-nested feature selection precluded claims of clinical utility. qPCR showed higher COL11A1 and SPON2 and lower PLA2G4A expression after TGF-β1 stimulation, whereas PITPNC1 did not differ significantly, providing model-specific support without establishing causal or clinical validity.

**Conclusion:**

Across the three cohorts, ECM remodeling was the most reproducible feature of AC, whereas RCD- and immune-related signals were cohort-dependent and did not support uniform activation of pyroptosis, ferroptosis, or necroptosis. The classifier and RCD signatures remain hypothesis-generating. The SW982 qPCR results provide model-specific experimental support for selected candidates, but broader functional validation in primary AC tissues and cells is required.

## 1 Introduction

Adhesive capsulitis (AC), commonly termed frozen shoulder, is a painful disorder characterized by progressive restriction of active and passive glenohumeral motion and capsular fibrosis. Its pathobiology is multifactorial and includes inflammatory signaling, fibroblast activation, altered extracellular-matrix (ECM) turnover, and subsequent tissue contracture. Diabetes mellitus and thyroid disease are established clinical associations, while genetic studies also support a heritable component to susceptibility (Dyer et al., 2023; Chuang et al., 2023; Kulm et al., 2022). Histologic and cellular studies of frozen-shoulder capsule have demonstrated activated fibroblast phenotypes, cytokine dysregulation, and enhanced matrix production, providing direct support for an inflammation–fibrosis axis in affected tissue (Akbar et al., 2019; Yang R. et al., 2022; Kim et al., 2025).

Recent single-cell work has refined this framework by showing that inflammatory and resolving macrophage states coexist with distinct fibroblast populations in frozen-shoulder capsule. In particular, MERTKlowCD48+ inflammatory macrophages and MERTK + LYVE1+MRC1+ macrophages enriched for negative regulators of inflammation were identified together with DKK3+ and POSTN + fibroblast populations, and integrin-mediated macrophage-fibroblast interactions were implicated in fibrosis resolution (Ng et al., 2024). These observations place immune-cell state and cell–matrix interactions at the center of AC biology and are consistent with the prominent ECM remodeling observed in bulk transcriptomic studies.

Regulated cell-death (RCD) is mechanistically linked to inflammation and tissue remodeling in other musculoskeletal disorders, but evidence in AC remains limited. Pyroptosis can amplify inflammatory signaling through gasdermin-mediated membrane permeabilization and cytokine release; ferroptosis is driven by iron-dependent lipid peroxidation; necroptosis involves RIPK1/RIPK3/MLKL signaling with the release of damage-associated molecular patterns. Experimental and patient studies in rheumatoid arthritis and osteoarthritis support the biological plausibility of these pathways in inflamed joint tissues (Zhang et al., 2023; Zhai et al., 2022; Chang et al., 2022; Liu et al., 2024; Chen et al., 2024; Liu et al., 2022; Yang J. et al., 2022). However, observations from analogous diseases cannot establish pathway execution in AC, and transcript abundance alone should not be interpreted as evidence of cell-death activation.

Prior AC bioinformatic studies have reported immune-related genes and candidate molecular signatures (Liu et al., 2023; Wen et al., 2024), but small cohorts, differing comparators, and analytical heterogeneity limit strong cross-study inference. Therefore, in the present study, we reanalyzed three independent public bulk RNA-seq cohorts without merging expression matrices across studies. We aimed to determine which transcriptional features replicate across cohorts; test whether curated pyroptosis, ferroptosis, and necroptosis signatures show consistent case–control behavior; characterize immune marker–gene associations without interpreting them as cell fractions; and evaluate an exploratory transcriptomic classifier. The primary discovery analysis explicitly adjusted for the substantial age and sex imbalance between AC cases and the non-healthy shoulder-instability comparator group. The manuscript additionally includes a qPCR experiment in TGF-β1-stimulated SW982 cells to evaluate four selected candidates in a downstream profibrotic model.

Additional tissue-level studies provide complementary evidence for an inflammation-to-fibrosis continuum in frozen shoulder. Classic histopathology described myofibroblast-rich capsular fibrosis with features resembling Dupuytren disease (Bunker and Anthony, 1995), while immunohistochemical analysis demonstrated synovial hyperplasia, type-III collagen deposition, and increased staining for fibrogenic and inflammatory cytokines (Rodeo et al., 1997). Reverse-transcription PCR studies subsequently documented altered expression of growth factors, cytokines, matrix metalloproteinases, and their inhibitors in frozen-shoulder capsule (Bunker et al., 2000), and later histologic work confirmed chronic inflammatory-cell infiltration together with fibroblast proliferation (Hand et al., 2007). MAPK/NF-kappaB signaling and expression of IL-6, MMP-3, and the related mediators have also been demonstrated in diseased synovium (Kanbe et al., 2009). Independent qPCR analyses reported increased inflammatory and fibrogenic cytokine transcripts (Kabbabe et al., 2010), whereas patient-derived glenohumeral synovial/capsular fibroblasts showed hyaluronan-sensitive regulation of procollagen and cytokine expression (Nago et al., 2010). Inflammatory cytokines are also elevated in the sub-acromial bursa of frozen shoulder (Lho et al., 2013). At the gene-expression level, a pilot study identified increased TNC and FN1 expression in AC tissue (Cohen et al., 2016), and a later multi-site analysis reported increased expression of several MMPs, IL6, SOX9, and COL1A1, supporting concurrent inflammatory, fibrotic, and chondrogenic remodeling (Nishimoto et al., 2022).

## 2 Materials and methods

### 2.1 Study design and public transcriptomic cohorts

This study was a secondary analysis of three independent public bulk RNA-sequencing cohorts of AC or frozen shoulder. Each cohort was processed and analyzed separately; expression matrices were not batch-corrected and merged across studies. GSE140731 was designated as the discovery cohort because it had the largest sample size and contained sample-level age and sex information. GSE190023 and GSE238053 were used as small independent cohorts for directional replication and exploratory transportability assessment rather than as definitive clinical validation cohorts.

GSE140731 included 48 surgical tissue samples: 22 from patients with AC and 26 from patients undergoing surgery for shoulder instability. Tissue was obtained from the anterior capsule and rotator interval. The comparator group was, therefore, a shoulder-instability surgical group rather than a healthy population. The AC and comparator groups also differed substantially in demographic composition: the mean age was 52.7 and 23.9 years, respectively, and women accounted for 15 of 22 AC samples and five of 26 comparator samples. These imbalances were incorporated into the primary discovery differential-expression and score models.

GSE190023 contained 12 proliferative synovium/joint-capsule samples, comprising four severe AC samples, four mild AC samples, and four healthy controls. The severe-AC-versus-control contrast was used for the principal validation display; mild-AC-versus-control, severe-AC-versus-mild-AC, and all-AC-versus-control contrasts were also examined. GSE238053 contained eight synovial samples, including four frozen-shoulder samples and four healthy controls. All samples represented in the verified count matrices were retained. Because matched covariate information was not available for the two small validation cohorts, their case–control contrasts were unadjusted.

The transcriptomic component used de-identified public data and did not involve new recruitment or access to identifiable human information. The cell-line experiment did not involve human participants or animals.

### 2.2 Data acquisition, sample annotation, and count-matrix audit

Raw gene-level count matrices and GEO sample metadata were obtained for GSE140731, GSE190023, and GSE238053. Sample titles were linked to their GEO sample accessions and then matched to the count-matrix columns. Group labels were verified against the series metadata before analysis. GSE140731 samples were classified as AC or shoulder-instability control; GSE190023 samples were classified as control, mild AC, or severe AC; and GSE238053 samples were classified as frozen shoulder or control.

Before preprocessing, every matrix was audited for missing values, negative entries, integer status, zero proportion, and sample alignment. The three matrices contained non-negative integer counts and were, therefore, treated as raw count data. No missing expression values were imputed. Per-sample library size was calculated as the sum of gene counts and was used for the sequencing-depth audit shown in Figure 1D.

**FIGURE 1:**
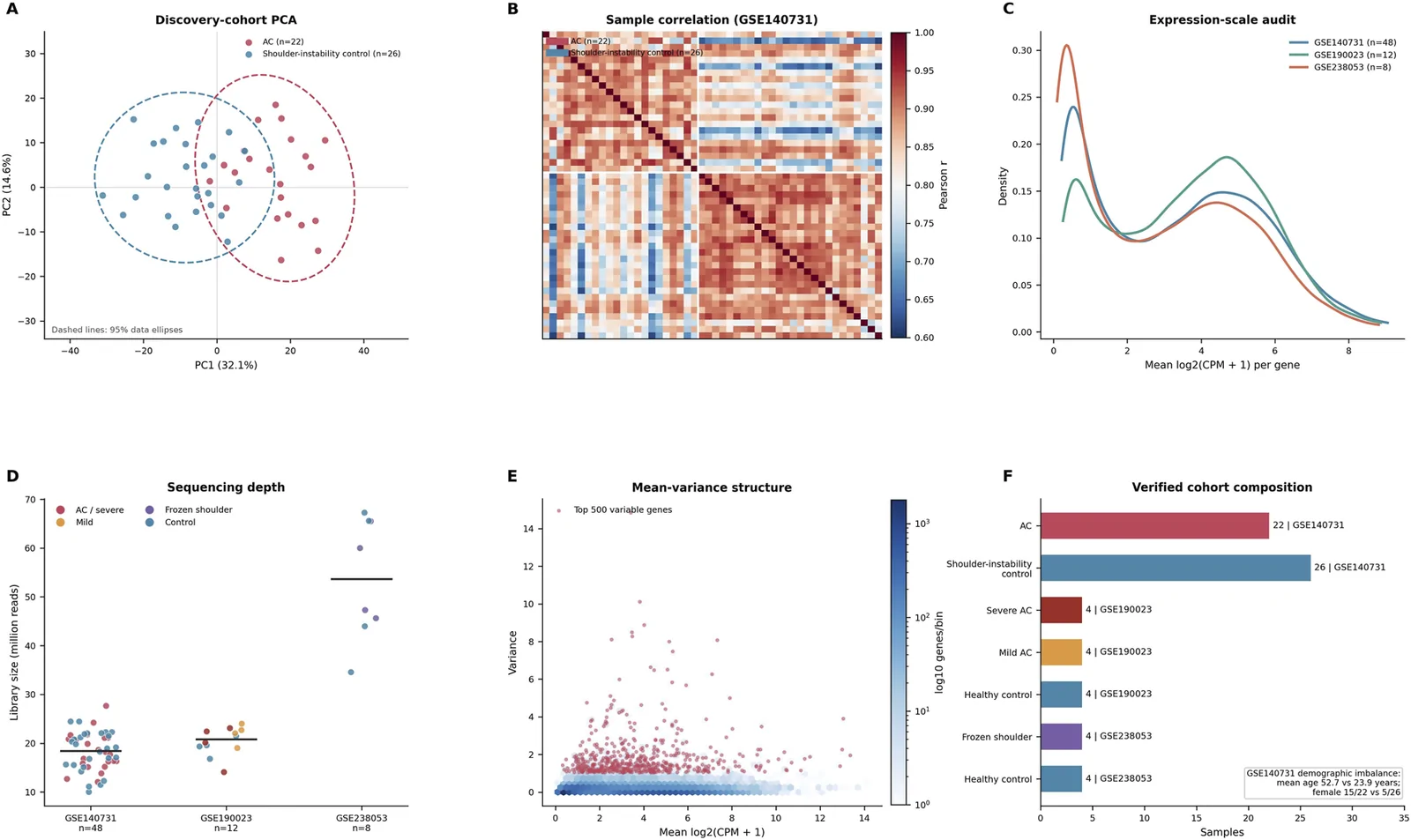
Cohort structure and expression data quality audit. **(A)** PCA of the 2,000 most variable genes in the GSE140731 discovery cohort. PC1 and PC2 explain 32.1% and 14.6% of variance, respectively. Dashed curves are 95% normal-theory data ellipses calculated from each group covariance matrix; they summarize dispersion and are not classification boundaries. **(B)** Sample–sample Pearson correlation matrix based on the same variable genes, ordered by phenotype. **(C)** Density distributions of mean gene expression on the log2(CPM +1) scale for GSE140731 (n = 48), GSE190023 (n = 12), and GSE238053 (n = 8). **(D)** Per-sample library sizes calculated from raw gene counts; horizontal lines show cohort medians. **(E)** Discovery-cohort mean-variance relationship on the log2(CPM +1) scale, with the 500 most variable genes highlighted. **(F)** Verified sample composition and GSE140731 demographic imbalance. GSE140731 controls were shoulder-instability surgical controls, not healthy controls.

### 2.3 Low-expression filtering, normalization, gene annotation, and quality-control visualization

Lowly expressed genes were removed separately within each cohort. A gene was retained when it had at least 10 counts in at least max [2, floor (0.10 × n)] samples, where n was the cohort sample size. Filtered raw counts were retained for count-aware differential-expression analysis. For visualization and score construction only, library-size normalization factors were calculated using the project-specific trimmed-mean-like implementation supplied with the analysis code. Counts were converted to counts per million (CPM), divided by the corresponding normalization factor, and transformed once as log2(CPM +1). This implementation was used consistently across cohorts but was not represented as the canonical edgeR TMM algorithm.

Ensembl gene identifiers were mapped to official gene symbols. Within-cohort visualization matrices retained one row per mapped symbol. For cross-cohort effect integration, duplicate symbols were resolved before merging by retaining the row with the highest DESeq2 baseMean; this prevented many-to-many joins and ensured that each symbol contributed once to concordance statistics.

Discovery-cohort principal component analysis (PCA) was performed on the 2,000 genes with the largest variance on the log2(CPM +1) scale. The revised Figure 1A uses 95% normal-theory data ellipses derived from each group covariance matrix and the chi-square distribution with two degrees of freedom; these ellipses are descriptive summaries of dispersion and are not classification boundaries. Sample–sample Pearson correlations were calculated from the same 2,000 variable genes. Additional audits included cohort-specific distributions of mean gene expression, per-sample library size, and the discovery-cohort mean-variance relationship; the 500 genes with the largest variance were marked separately.

### 2.4 Differential-expression analysis

Differential expression was estimated from filtered raw counts using PyDESeq2. Effect estimates were oriented as case minus control, so a positive log2 fold change indicated higher expression in the AC/frozen-shoulder group. In GSE140731, age was standardized across all samples and the primary design was specified as ∼ age_z + sex + group. The reported group coefficient, therefore, represents the AC-versus-shoulder-instability difference after adjustment for age and sex. Cook-distance refitting was enabled. In GSE190023, separate unadjusted models were fitted for severe AC versus control, mild AC versus control, severe AC versus mild AC, and all AC versus control. GSE238053 was analyzed as frozen shoulder versus control.

P-values were adjusted within each differential-expression contrast by the Benjamini–Hochberg procedure. Genes with a false-discovery rate (FDR) q < 0.05 were considered differentially expressed; no additional absolute fold-change threshold was imposed. Volcano plots show nominal P-values on the vertical axis and FDR significance by color. Cumulative FDR profiles counted the number of genes meeting thresholds from 1 × 10^−8^ to 0.05.

### 2.5 Regulated cell-death gene sets, gene-level effects, and signature scores

Three RCD programs were evaluated: pyroptosis, ferroptosis, and necroptosis. The pyroptosis score used a prespecified nine-gene core set (GSDMD, CASP1, NLRP3, IL1B, IL18, PYCARD, AIM2, GSDME, and CASP4). Ferroptosis and necroptosis sets were derived from KEGG_2021_Human and supplemented with canonical core genes. The ferroptosis set included GPX4, SLC7A11, ACSL4, TF, TFRC, and HMOX1 among its core members; the necroptosis set included RIPK1, RIPK3, MLKL, FADD, and CASP8 among its core members. Only genes quantified in a given cohort contributed to that cohort score, and the number of contributing genes was retained in the output.

For each cohort and each RCD program, the expression of every constituent gene was standardized across all samples in that cohort, and the sample-level signature score was calculated as the mean of the available gene-wise z-scores. These scores summarize relative transcript abundance; they do not directly measure gasdermin pore formation, lipid peroxidation, or RIPK3/MLKL-dependent cell-death execution. Figures 2F, 3B display individual-gene log2 fold changes and FDR calls and were not used as evidence that an entire RCD pathway was activated.

**FIGURE 2:**
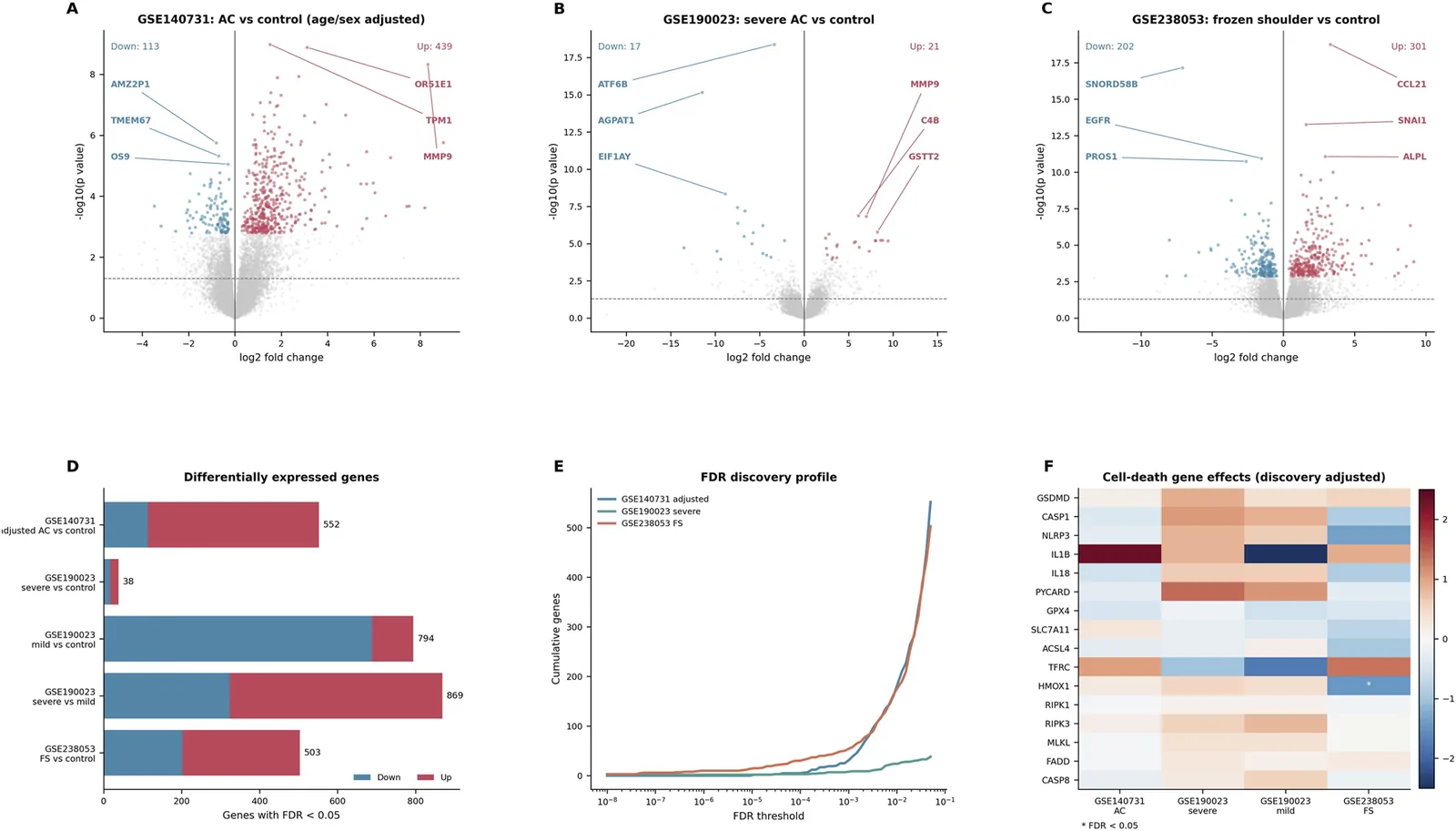
Differential-expression landscape across three adhesive capsulitis cohorts. **(A)** Volcano plot for the age- and sex-adjusted GSE140731 AC-versus-shoulder-instability contrast (439 upregulated and 113 downregulated genes at FDR q < 0.05). **(B)** GSE190023 severe-AC-versus-healthy-control contrast (21 upregulated and 17 downregulated genes). **(C)** GSE238053 frozen-shoulder-versus-healthy-control contrast (301 upregulated and 202 downregulated genes). Points are colored by FDR significance; no absolute log2-fold-change cutoff was imposed. **(D)** Numbers of FDR-significant upregulated and downregulated genes across the prespecified contrasts. **(E)** Cumulative number of significant genes across FDR thresholds from 1 × 10^−8^ to 0.05. **(F)** Log2 fold changes of selected pyroptosis-, ferroptosis-, and necroptosis-related genes; asterisks indicate FDR q < 0.05. The GSE140731 column is age/sex adjusted. Panel F is a gene-level direction map and does not measure whole-pathway activation.

**FIGURE 3:**
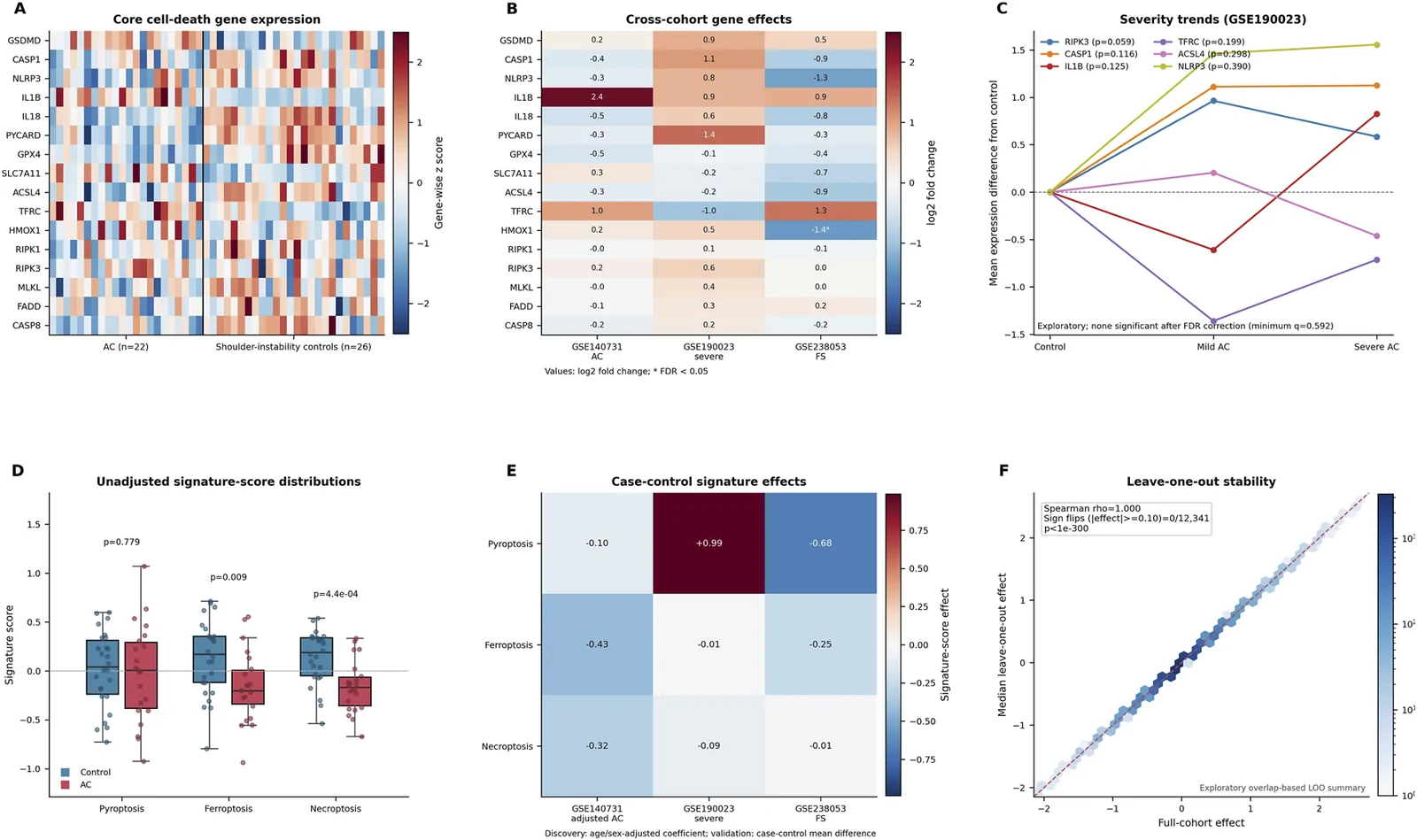
Regulated cell-death genes, signature scores, and stability boundaries. **(A)** Gene-wise z-scored expression of core RCD genes across GSE140731 samples. **(B)** Cross-cohort log2 fold changes for the same genes; the GSE140731 effect is age/sex adjusted, and asterisks indicate FDR q < 0.05. **(C)** Mean expression differences from control across control, mild AC, and severe AC in GSE190023 for the six most variable displayed genes; none remained significant after multiplicity correction (minimum adjusted value = 0.592). **(D)** Unadjusted discovery distributions of pyroptosis, ferroptosis, and necroptosis scores, with Welch-test P-values. **(E)** Case–control score effects: age/sex-adjusted coefficients for GSE140731 and unadjusted mean differences for the validation cohorts. Ferroptosis and necroptosis point estimates were non-positive across cohorts, but validation estimates were small and imprecise; pyroptosis directions varied. **(F)** Full-cohort versus median leave-one-out gene expression effects in GSE140731. The near-unity correlation is expected because leave-one-out datasets overlap extensively and is presented only as an exploratory stability check.

Unadjusted discovery score distributions were compared with Welch two-sample t-tests. For the primary GSE140731 analysis, each signature score was modeled by ordinary least squares with the case status, standardized age, and sex as predictors; heteroscedasticity-consistent HC3 standard errors were used, and the three signature P*-*values were adjusted by the Benjamini–Hochberg method. In GSE190023 and GSE238053, validation effects were expressed as unadjusted case–control mean differences. Severity trends in GSE190023 were explored across the control, mild AC, and severe AC with Kruskal–Wallis tests and multiplicity adjustment across the tested genes.

As an exploratory stability analysis in GSE140731, the full-cohort case–control mean difference was compared with the median effect obtained after leaving out one sample at a time. Spearman correlation and the number of sign changes were reported. Because successive leave-one-out datasets shared nearly all samples, a very high correlation was expected by construction and was not interpreted as independent replication.

### 2.6 Functional enrichment and leading-edge analysis

Over-representation analysis (ORA) was used as the descriptive annotation of the original unadjusted GSE140731 contrast. The 300 most significant FDR-positive upregulated genes and the 300 most significant FDR-positive downregulated genes were analyzed separately with the GO Biological Process 2023 and KEGG 2021 Human libraries through GSEApy/Enrichr. The displayed adjusted P-values were those returned by the enrichment analysis.

The primary pathway analysis used preranked gene-set enrichment analysis based on the age- and sex-adjusted GSE140731 DESeq2 Wald statistic after enforcing one row per gene symbol. Gene sets containing 10–500 quantified genes were tested with 1,000 permutations and a fixed random seed of 42. KEGG pathways were analyzed together with prespecified ferroptosis and necroptosis sets. The nine-gene pyroptosis core set did not meet the minimum size of 10 for this preranked analysis and was, therefore, evaluated through the gene-level and score-based analyses rather than the preranked analysis shown in Figure 4D. The normalized enrichment score (NES), nominal P-value, and FDR q value were reported. Leading-edge genes were extracted from selected FDR-significant pathways.

**FIGURE 4:**
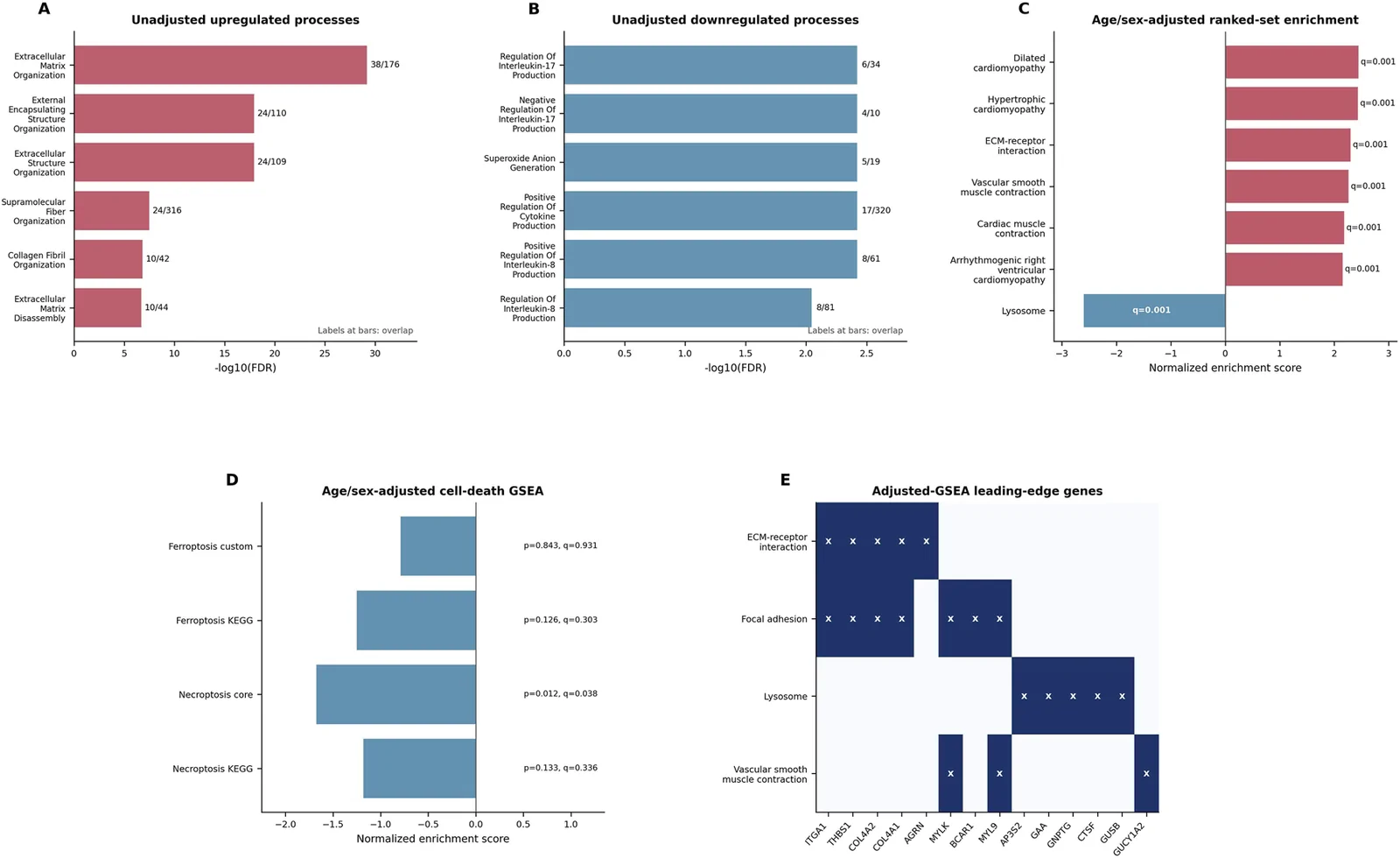
Pathway enrichment and leading-edge analysis. **(A)** Descriptive over-representation analysis of upregulated genes from the unadjusted GSE140731 contrast, showing prominent extracellular matrix and collagen-related biological processes. **(B)** Descriptive over-representation analysis of downregulated discovery genes. Bars in A and B show -log10 adjusted P-values, and labels indicate overlap/input-set counts. **(C)** Age- and sex-adjusted preranked gene-set enrichment analysis based on the GSE140731 DESeq2 Wald statistic. ECM–receptor interaction was positively enriched (NES = 2.299, q = 0.001), whereas lysosome was negatively enriched. **(D)** Adjusted RCD gene-set results. The core necroptosis set was negatively enriched (P = 0.012, q = 0.038); the displayed ferroptosis sets and KEGG necroptosis were not FDR-significant. **(E)** Leading-edge membership for selected adjusted pathways.

### 2.7 Immune and stromal marker–gene scoring

Fifteen curated marker panels were used to summarize B cells, plasma cells, CD8 T cells, CD4 T cells, regulatory T cells, natural killer (NK) cells, monocytes, M1-like macrophages, M2-like macrophages, dendritic cells, mast cells, neutrophils, endothelial cells, fibroblasts, and smooth muscle cells. Marker panels were assembled from commonly used LM22- and Bindea-derived gene lists. For each panel, genes quantified in the cohort were standardized across samples and averaged to obtain a sample-level global-z marker score. A panel was retained when at least two markers were available. These values are marker–gene summaries and not deconvolution-derived cell fractions or direct cell-abundance estimates.

Discovery-cohort marker scores were modeled with case status, standardized age, and sex using ordinary least squares with HC3 standard errors. P-values were adjusted across the 15 panels. Validation-cohort comparisons used Welch tests within cohort, followed by cohort-wise Benjamini–Hochberg adjustment. Sample-level heatmaps were generated from the marker scores. Exploratory Spearman correlations between selected RCD genes and marker scores were calculated after removing the queried RCD gene from a marker panel whenever the same gene occurred in both sets, thereby avoiding tautological self-correlation. Figure 5F counts the overlap between discovered differentially expressed genes and immune-related annotation sets; it is descriptive and is not a formal enrichment test.

**FIGURE 5:**
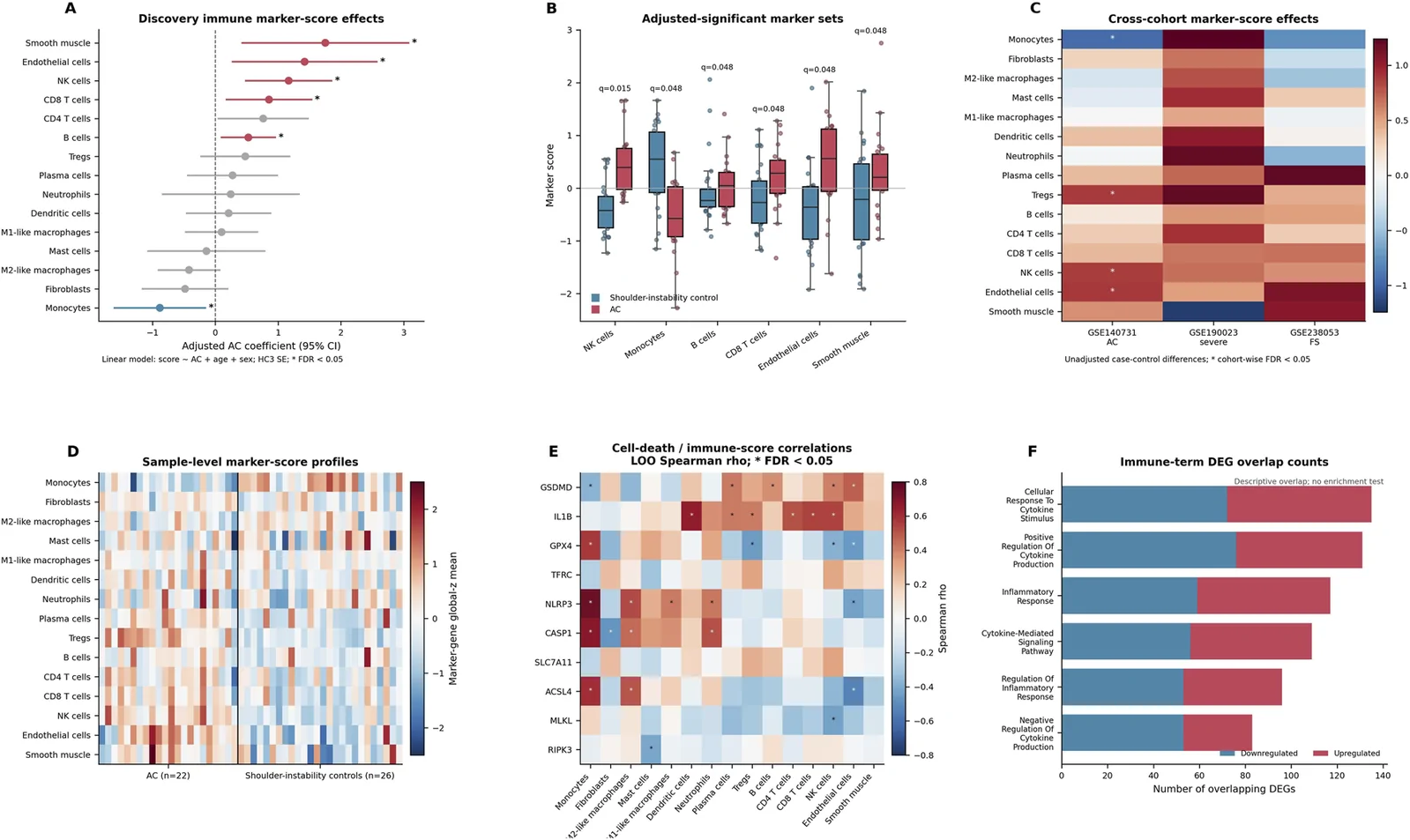
Immune and stromal marker score analyses. **(A)** Age- and sex-adjusted GSE140731 marker score coefficients with 95% confidence intervals. Smooth muscle, endothelial cell, NK cell, CD8 T cell, and B cell scores were higher in AC, whereas the monocyte score was lower; six panels met FDR q < 0.05. **(B)** Discovery cohort distributions of the six FDR-significant scores. **(C)** Unadjusted case–control marker score effects across GSE140731, GSE190023, and GSE238053; no marker panel met cohort-wise FDR significance in either validation cohort. **(D)** Sample-level marker score heatmap for GSE140731. **(E)** Exploratory Spearman correlations between selected RCD genes and marker scores after removal of overlapping marker genes; asterisks indicate FDR q < 0.05. **(F)** Counts of discovery differentially expressed genes overlapping immune-related annotation terms. Panels A–E use marker–gene global-z scores, not deconvolution-derived cell fractions, and panel F is descriptive rather than a formal enrichment test.

### 2.8 Exploratory three-gene classifier

The classifier was developed as an exploratory transcriptomic separation model, not as a clinical diagnostic test. The candidate pool comprised quantified RCD-related genes together with the 100 highest-ranked discovered differentially expressed genes not already represented in the RCD set. An L1-penalized logistic regression was evaluated over 50 values of the inverse regularization parameter C from 0.001 to 10. Five-fold stratified cross-validation with shuffling and a fixed random seed of 42 was used to select C according to the mean validation-fold AUC. Standardization was performed within the model-development workflow, and pooled out-of-fold probabilities were used for the GSE140731 ROC curve; the apparent resubstitution AUC was not reported.

At the selected C, the genes retained and quantified in all three cohorts were COL11A1, SPON2, and PITPNC1. A fixed three-gene logistic score was fitted in the discovery cohort and assessed in GSE190023 (all AC versus control and severe AC versus control) and GSE238053 (frozen shoulder versus control). Cohort-level ROC AUCs were accompanied by percentile 95% confidence intervals from 1,000 bootstrap resamples; resamples containing only one outcome class were discarded. Because the discovery candidate pool was defined before cross-validation and the validation cohorts were very small, the entire classifier analysis remained non-nested and exploratory even after replacing the training AUC with out-of-fold performance.

A random forest with 1,000 trees and maximum depth five was fitted to the discovery candidate pool to provide a descriptive feature-importance ranking; it was not treated as an independent feature-selection or validation procedure. To avoid the earlier metric inconsistency, the revised Figures 6D, 7A/C use the same one-symbol-per-gene DESeq2 log2 fold-change estimates: age- and sex-adjusted effects for GSE140731 and unadjusted effects for GSE190023 and GSE238053.

**FIGURE 6:**
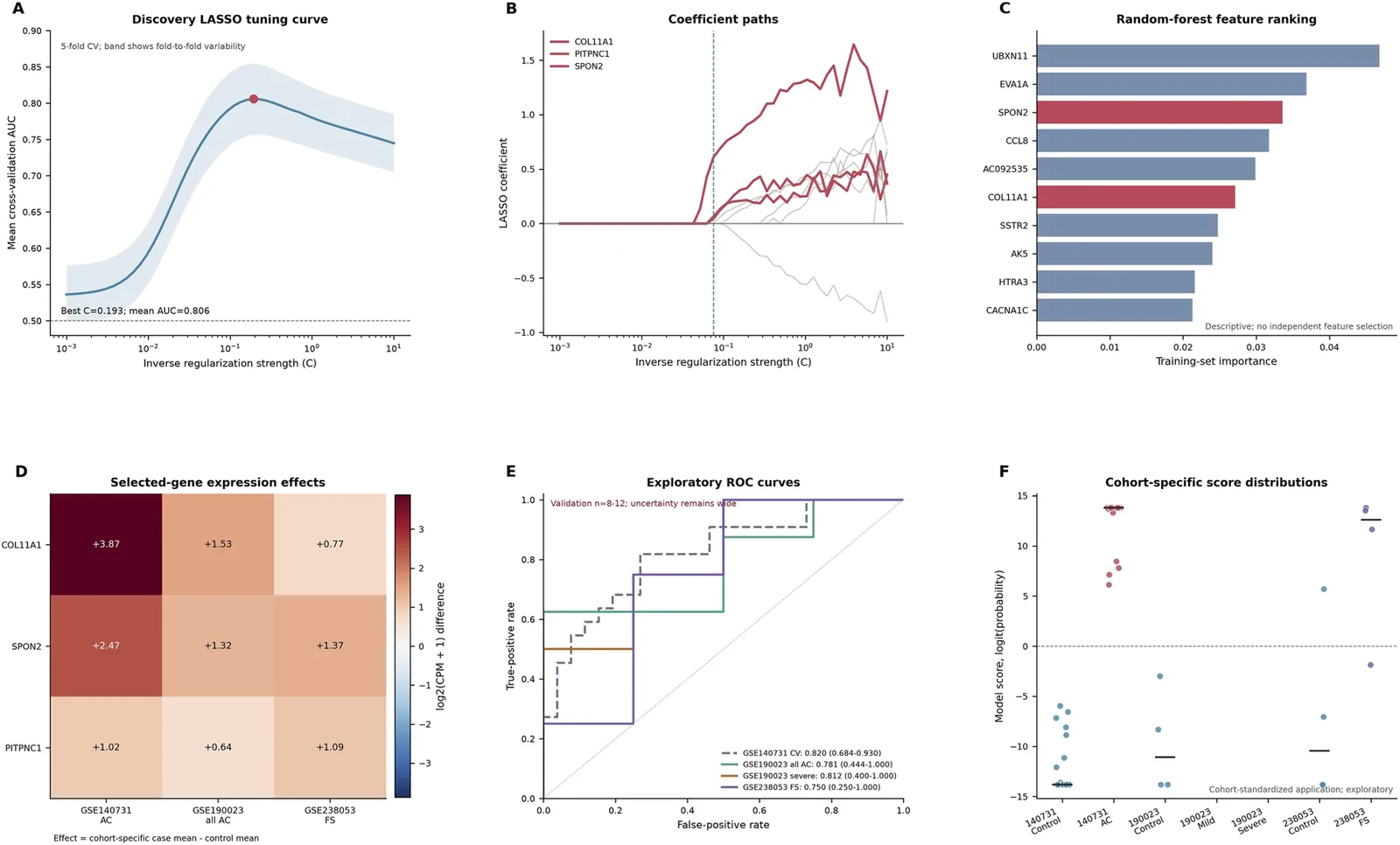
Exploratory COL11A1–SPON2–PITPNC1 classifier. **(A)** Mean five-fold validation AUC across the L1-logistic C grid; the selected value was C = 0.193, with mean fold AUC = 0.806. The band shows variation across folds. **(B)** L1 coefficient paths across the regularization sequence. **(C)** Descriptive random-forest feature-importance ranking; this analysis was not used as an independent validation step. **(D)** Cross-cohort unique symbol DESeq2 log2 fold changes for COL11A1, SPON2, and PITPNC1, using the same effect definition as Figure 7A/C: age/sex-adjusted GSE140731 estimates and unadjusted validation estimates. **(E)** ROC curves based on pooled out-of-fold probabilities for GSE140731 and fixed-score evaluation in the small external cohorts. AUCs were 0.820 (95% CI, 0.684–0.930), 0.781 (0.444–1.000), 0.812 (0.400–1.000), and 0.750 (0.250–1.000) for GSE140731, GSE190023 all AC, GSE190023 severe AC, and GSE238053, respectively. **(F)** Cohort-specific three-gene score distributions. Because candidate preselection was not fully nested and validation cohorts were small, the model is exploratory and is not a clinical diagnostic model.

**FIGURE 7:**
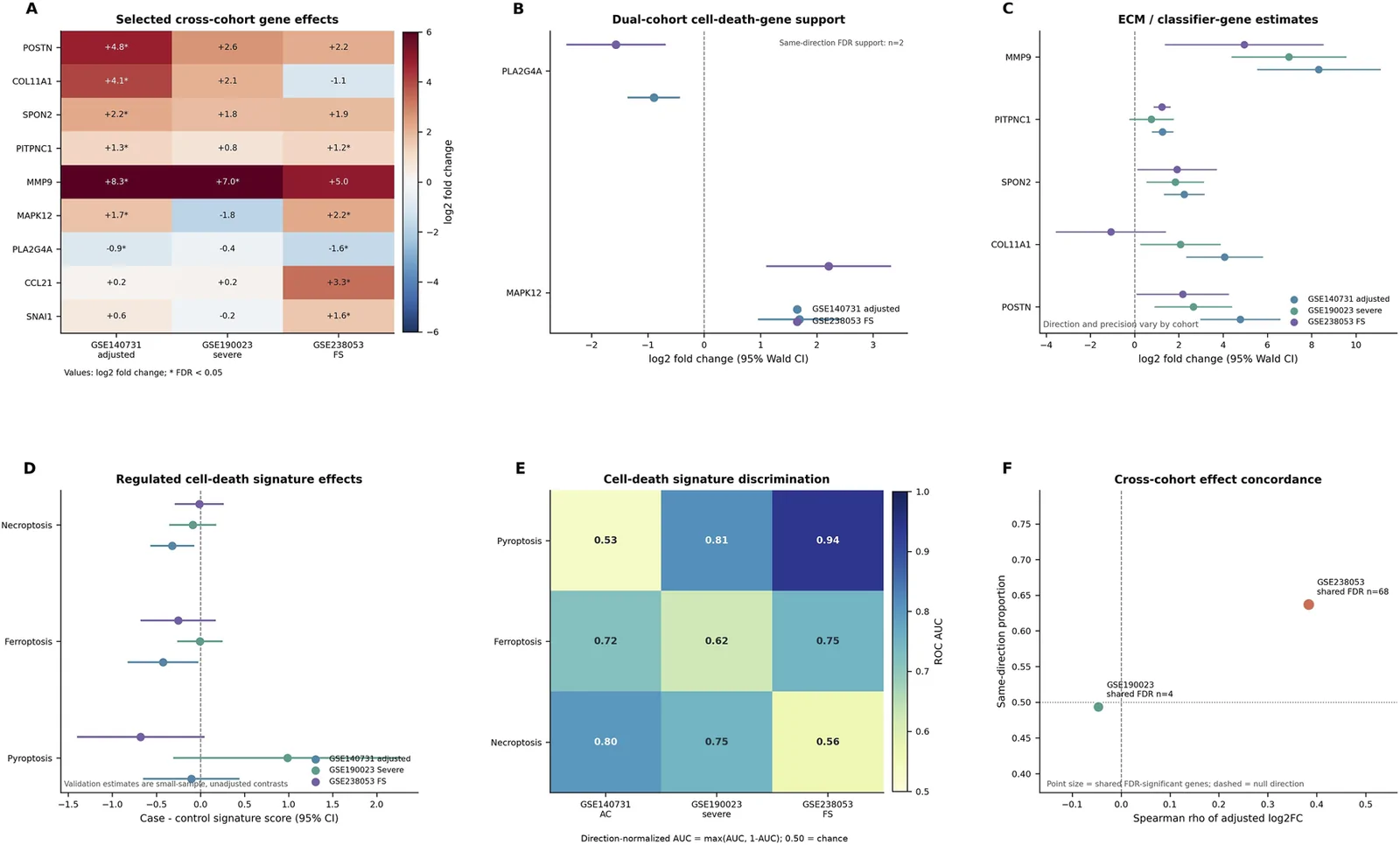
Cross-cohort synthesis and quantitative validation boundaries. **(A)** Unique-symbol DESeq2 log2 fold changes for selected ECM-, classifier-, inflammatory-, and RCD-related genes across GSE140731, GSE190023, and GSE238053. GSE140731 is age/sex-adjusted; validation estimates are unadjusted. **(B)** RCD-related genes with same-direction FDR significance in two cohorts: PLA2G4A was downregulated and MAPK12 was upregulated in GSE140731 and GSE238053. **(C)** Forest plot of selected gene effects with 95% confidence intervals, showing stable positive MMP9 effects and a negative COL11A1 effect in GSE238053. **(D)** RCD signature-score effects with 95% confidence intervals; validation intervals frequently cross zero. **(E)** Direction-normalized AUCs, defined as max (AUC, 1 - AUC), for pyroptosis, ferroptosis, and necroptosis scores. Values quantify *post hoc* separation magnitude only; direction is shown in panel D, and no diagnostic claim is made. **(F)** Integrated effect concordance: the x-axis shows Spearman rho, the y-axis shows same-direction proportion, and point size reflects the number of genes significant in both cohorts. Dashed lines mark rho = 0 and 50% direction agreement.

### 2.9 STRING network analysis and cross-cohort effect concordance

The network input comprised the three classifier genes and discovered RCD-related genes meeting FDR significance. Protein–protein associations were queried from STRING version 11.5 for *Homo sapiens* with a required combined score of at least 0.400. Self-loops were removed, reciprocal edges were converted to a single undirected pair, and the highest score was retained when duplicate pairs occurred. Node strength was defined as the sum of STRING confidence scores across all incident edges.

Within GSE140731, Spearman correlations were calculated between network hub genes and quantified RCD genes on the log2(CPM +1) scale. Hub–target pairs in which the same gene appeared on both axes were excluded, and correlation P-values were adjusted for multiple testing. Cross-cohort concordance was calculated after enforcing one DESeq2 row per gene symbol. The age- and sex-adjusted GSE140731 log2 fold changes were compared separately with unadjusted all-AC-versus-control effects in GSE190023 and frozen-shoulder-versus-control effects in GSE238053. Spearman rho, the proportion of genes with the same sign, the number of genes significant at FDR < 0.05 in both cohorts, and the sign agreement among the shared significant genes were recorded.

### 2.10 Direction-normalized discrimination of RCD scores

For each RCD score and cohort, a descriptive ROC AUC was calculated from the corresponding case–control comparison. To summarize the separation magnitude without implying a uniform biological direction, the displayed value was direction-normalized as max (AUC, 1 - AUC). Thus, an AUC above 0.50 indicates only that the score separates the two groups after allowing the direction to be chosen *post hoc*; it does not show whether the score is higher or lower in cases and does not constitute external diagnostic validation. Direction and uncertainty were evaluated separately from the case–control effect estimates in Figures 3E, 7D.

### 2.11 SW982 cell culture, TGF-β1-induced profibrotic stimulation, RNA extraction, and quantitative real-time PCR

SW982 human synovial sarcoma cells were obtained from Wuhan Pricella Biotechnology Co., Ltd. (Procell, Wuhan, China; cat. no. CL-0450). The supplier-provided short tandem repeat (STR) authentication profile was reviewed to confirm cell-line identity, and cultures were confirmed to be free of *mycoplasma* before experimentation using the MycAway Plus-Color One-Step *Mycoplasma* Detection Kit (2G; Yeasen Biotechnology, Shanghai, China; cat. no. 40615ES). Cells were maintained in Leibovitz’s L-15 medium (Procell; cat. no. PM151010) supplemented with 10% fetal bovine serum (Procell; cat. no. 164210-50) and 1% penicillin–streptomycin (Procell; cat. no. PB180120) at 37 °C in a humidified ambient-air incubator without supplemental CO_2_. Cultures were passaged at 70%–80% confluence using 0.25% trypsin-EDTA (Procell; cat. no. PB180225), and passages 3–8 after thawing were used.

For stimulation, 2.0 × 10^5^ cells were seeded in each well of six-well plates in 2 mL complete medium and allowed to attach for 24 h. The medium was then replaced with L-15 containing 1% fetal bovine serum for 12 h, after which the cells were exposed to 10 ng/mL recombinant human TGF-β1 (Novoprotein, Suzhou, China; cat. no. CA59) for 24 h. This condition was defined operationally as a TGF-β1-induced profibrotic AC-like stimulus; vehicle-control wells received the same volume of sterile water. The experiment was performed in three separate experimental runs. In each run, two independently seeded and processed wells were included per condition, yielding six independent culture replicates per group. Each well was lysed and analyzed separately and constituted the unit of statistical analysis. Each cDNA sample was analyzed in three technical qPCR wells, and technical Ct values were averaged before the calculation of relative expression; technical wells were not treated as independent replicates.

Total RNA was extracted using the FastPure Cell/Tissue Total RNA Isolation Kit V2 (Vazyme Biotech, Nanjing, China; cat. no. RC112-01) according to the manufacturer’s instructions. RNA concentration and purity were measured with a Nano-500 micro-spectrophotometer (Allsheng Instruments, Hangzhou, China); samples with A260/A280 ratios of 1.8–2.1 and A260/A230 ratios of at least 1.8 were used. For each sample, 1.0 μg total RNA was subjected to genomic-DNA removal at 42 °C for 2 min and reverse-transcribed in a 20-μL reaction using HiScript III RT SuperMix for qPCR (+gDNA wiper; Vazyme; cat. no. R323-01) at 37 °C for 15 min followed by 85 °C for 5 s. Quantitative real-time PCR was performed on a LineGene 9600 Plus fluorescence quantitative PCR system (FQD-96A; Bioer Technology, Hangzhou, China) using ChamQ Universal SYBR qPCR Master Mix (Vazyme; cat. no. Q711-02). Each 20-μL reaction contained 10 μL of 2× master mix, 0.4 μL each of 10 μM forward and reverse primers, 1.0 μL diluted cDNA, and 8.2 μL nuclease-free water. Cycling conditions were 95 °C for 30 s, followed by 40 cycles of 95 °C for 10 s and 60 °C for 30 s; a 65 °C–95 °C melting-curve analysis was then performed. GAPDH was used as the reference gene. Primers were synthesized by Sangon Biotech (Shanghai, China), and their sequences are listed in Table 1. No-template and no-reverse-transcription controls were included in each run, and amplification specificity was accepted only when a single melting-curve peak was observed.

**TABLE 1:** Primer sequences used for quantitative real-time PCR.

Gene	Forward primer (5′-3′)	Reverse primer (5′-3′)
COL11A1	ATG​GAC​CAG​CAG​GAT​TAC​GTG​G	TGT​ACC​TGC​TGA​CCC​ACG​TTC​T
SPON2	GAT​TGT​AGA​CAG​CGC​CTC​AGT​TCC	GAC​GCA​CTC​AGC​CTC​TTC​TTC​G
PITPNC1	GTC​CGA​GAC​ATT​CTG​CTG​ATT​GG	CTT​GTT​GGT​GGC​TTC​CTG​AGT​G
PLA2G4A	GGA​TTC​TCT​GGT​GTG​ATG​AAG​GC	CCT​TTC​TCT​GGA​AAA​TCA​GGG​TG
GAPDH	GTC​TCC​TCT​GAC​TTC​AAC​AGC​G	ACC​ACC​CTG​TTG​CTG​TAG​CCA​A

Relative expression was calculated using the 2^−ΔΔCT^ method, with GAPDH as the internal reference and the mean DeltaCt of the vehicle-control group as the calibrator. Four genes were evaluated: COL11A1, SPON2, and PITPNC1 from the exploratory classifier and PLA2G4A as an RCD-related gene with same-direction significance in GSE140731 and GSE238053. The six independent culture-replicate values per condition were compared using two-sided Welch t tests. The qPCR experiment was interpreted as model-specific expression validation; it did not measure protein abundance or the execution of pyroptosis, ferroptosis, or necroptosis.

### 2.12 Statistical analysis and reproducibility

All tests were two-sided. Continuous transcriptomic score comparisons used Welch tests, unless an adjusted linear model was specified. Age- and sex-adjusted score analyses used HC3 heteroscedasticity-consistent standard errors. Kruskal–Wallis tests were used for the exploratory three-level severity analysis. Benjamini–Hochberg FDR correction was applied within prespecified families of differential-expression, score, enrichment, and correlation tests, unless otherwise indicated. FDR q < 0.05 was considered statistically significant. Effect sizes, confidence intervals, and exact P*-* or q-values were reported whenever available; small validation-cohort estimates were interpreted with their wide uncertainty.

Analyses were performed in Python using pandas, NumPy, SciPy, statsmodels, scikit-learn, PyDESeq2, GSEApy, and NetworkX; figures were generated with Matplotlib. Randomized procedures used a fixed seed of 42. The three cohorts were analyzed independently, and all cross-cohort integrations used one row per gene symbol.

## 3 Results

### 3.1 Cohort composition and expression-data quality

The analysis included 68 samples across three cohorts. GSE140731 comprised 22 AC samples and 26 shoulder-instability surgical controls; GSE190023 comprised four severe AC, four mild AC, and four healthy-control samples; and GSE238053 comprised four frozen-shoulder and four healthy-control samples. The discovery comparator was not healthy and was substantially younger and included more males than the AC group (mean age, 23.9 versus 52.7 years; women, 5/26 versus 15/22), which motivated age- and sex-adjusted primary discovery analyses (Figure 1F).

PCA of the 2,000 most variable genes in GSE140731 showed a case–control separation trend rather than complete separation. PC1 and PC2 explained 32.1% and 14.6% of total variance, respectively, and several AC and shoulder-instability samples occupied overlapping regions of the score plot (Figure 1A). The corrected 95% data ellipses summarized within-group dispersion without treating the ellipse boundary as a classification rule. The sample-correlation heatmap similarly showed generally high within-group correlation but also substantial cross-group correlation, indicating that neither group was transcriptionally homogeneous (Figure 1B).

All cohorts were displayed on the same mean log2(CPM +1) scale. The density distributions differed among datasets but showed no evidence of a second logarithmic transformation (Figure 1C). GSE140731 and GSE190023 libraries contained approximately 10–28 million assigned gene counts per sample, whereas GSE238053 libraries were larger, at approximately 35–67 million (Figure 1D). The discovery cohort retained a pronounced mean-variance relationship, with many low-expression genes and a distinct high-variance tail; the 500 most variable genes were marked separately (Figure 1E).

### 3.2 Differential-expression landscapes differed markedly across cohorts

After adjustment for age and sex, the GSE140731 AC-versus-shoulder-instability contrast identified 552 FDR-significant genes, including 439 upregulated and 113 downregulated genes (Figure 2A). Representative upregulated genes included OR51E1, TPM1, and MMP9, whereas AMZ2P1, TMEM67, and OS9 were among the downregulated genes. The direction and number of significant genes were, therefore, not attributable solely to an unadjusted case–control comparison, although residual confounding by the non-healthy comparator could not be excluded.

The smaller validation cohorts produced different discovery scales. In GSE190023, severe AC versus healthy control yielded 38 FDR-significant genes (21 upregulated and 17 downregulated), with MMP9, C4B, and GSTT2 among the positive effects and ATF6B, AGPAT1, and EIF1AY among the negative effects (Figure 2B). Mild AC versus control yielded 794 significant genes, and severe AC versus mild AC yielded 869. In GSE238053, frozen shoulder versus healthy control identified 503 significant genes (301 upregulated and 202 downregulated); CCL21, SNAI1, and ALPL were upregulated, whereas SNORD58B, EGFR, and PROS1 were downregulated (Figures 2C,D).

Cumulative FDR profiles showed rapid increases in the number of significant genes as the threshold was relaxed in GSE140731 and GSE238053, whereas the GSE190023 severe-versus-control contrast remained comparatively sparse (Figure 2E). The RCD-focused heatmap in Figure 2F shows gene-specific positive, negative, and non-significant effects across cohorts. Because it contains selected individual genes rather than a pathway-level statistic, this panel was interpreted only as a map of gene-level direction and did not establish activation or suppression of pyroptosis, ferroptosis, or necroptosis as complete programs.

### 3.3 RCD-related genes and scores did not support uniform pathway activation

Core RCD genes varied across discovery samples, but the heatmap did not show a coherent pattern in which all genes belonging to one pathway changed in the same direction (Figure 3A). Cross-cohort gene effects were also heterogeneous (Figure 3B). For example, HMOX1 was reduced by approximately 1.4 log2 fold in GSE238053 and reached FDR significance, whereas its magnitude and significance differed in the other cohorts. These gene-level differences supported the evaluation of aggregate scores but did not, by themselves, indicate pathway execution.

In GSE190023, RIPK3, CASP1, IL1B, TFRC, ACSL4, and NLRP3 showed different mean trajectories from control to mild and severe AC, but none remained significant after multiplicity correction; the smallest adjusted value was 0.592 (Figure 3C). The severity panel was, therefore, treated as descriptive and did not support a monotonic stage-dependent RCD response.

Unadjusted GSE140731 score distributions showed no difference in pyroptosis (P = 0.779), lower ferroptosis scores in AC (P = 0.009), and lower necroptosis scores in AC (P = 4.4 × 10^−4^ Figure 3D). After adjustment for age and sex, the discovery effect estimates were −0.10 for pyroptosis, −0.43 for ferroptosis, and −0.32 for necroptosis. The corresponding FDR results were non-significant for pyroptosis (q = 0.708), borderline for ferroptosis (q = 0.056), and significant for necroptosis (q = 0.035).

Across the three cohorts, the adjusted/unadjusted point estimates were −0.10, +0.99, and −0.68 for pyroptosis; −0.43, −0.01, and −0.25 for ferroptosis; and −0.32, −0.09, and −0.01 for necroptosis in GSE140731, GSE190023, and GSE238053, respectively (Figure 3E). Thus, ferroptosis and necroptosis point estimates were consistently non-positive rather than directionally conflicting, but the effects were close to zero and imprecise in the small validation cohorts, and formal significance did not replicate. Pyroptosis showed clearly different directions across cohorts. Collectively, the score analyses did not support a claim of uniform RCD activation in AC.

The full-cohort expression effects and median leave-one-out effects were almost perfectly correlated (Spearman rho = 1.000), with no sign changes among 12,341 informative genes (Figure 3F). Because each leave-one-out analysis retained 47 of 48 discovery samples, this near-unity correlation was structurally expected and was considered an exploratory internal-stability check rather than independent validation.

### 3.4 ECM-related enrichment was the dominant adjusted pathway-level signal

Descriptive ORA of unadjusted discovered upregulated genes was dominated by extracellular-structure terms (Figure 4A). Extracellular matrix organization contained 38 of 176 input genes, external encapsulating structure organization contained 24 of 110, extracellular structure organization contained 24 of 109, supramolecular fiber organization contained 24 of 316, collagen fibril organization contained 10 of 42, and extracellular matrix disassembly contained 10 of 44. In contrast, downregulated-gene annotations included regulation of interleukin-17 production (6/34), negative regulation of interleukin-17 production (4/10), superoxide anion generation (5/19), positive regulation of cytokine production (17/320), positive regulation of interleukin-8 production (8/61), and regulation of interleukin-8 production (8/81; Figure 4B).

The age- and sex-adjusted preranked analysis provided the principal pathway result (Figure 4C). Dilated cardiomyopathy, hypertrophic cardiomyopathy, ECM–receptor interaction, vascular smooth muscle contraction, cardiac muscle contraction, and arrhythmogenic right ventricular cardiomyopathy showed positive enrichment, whereas lysosome showed negative enrichment. ECM–receptor interaction had an NES of 2.299 and remained significant after FDR correction (q = 0.001), making ECM-related remodeling the clearest pathway-level signal in the adjusted discovery analysis.

The RCD-focused enrichment results did not show a parallel positive activation pattern (Figure 4D). The custom ferroptosis set was non-significant (P = 0.843, q = 0.931), as was KEGG ferroptosis (P = 0.126, q = 0.303). The core necroptosis set was negatively enriched (P = 0.012, q = 0.038), whereas KEGG necroptosis was not FDR-significant (P = 0.133, q = 0.336). Leading-edge genes included ITGA1, THBS1, COL4A2, COL4A1, and AGRN for ECM–receptor interaction; ITGA1, THBS1, COL4A2, COL4A1, MYLK, BCAR1, and MYL9 for focal adhesion; AP3S2, GAA, GNPTG, CTSF, and GUSB for lysosome; and MYLK, MYL9, and GUCY1A2 for vascular smooth muscle contraction (Figure 4E).

### 3.5 Immune and stromal marker-score associations were confined to the discovery cohort

After adjustment for age and sex in GSE140731, six of the 15 marker scores met FDR significance (Figure 5A). Smooth muscle, endothelial cell, NK cell, CD8 T cell, and B cell scores were higher in AC, whereas the monocyte score was lower. The largest reported positive coefficients were 1.421 for endothelial cells and 1.168 for NK cells, while the monocyte coefficient was −0.883. The NK-cell score had q = 0.015; the other significant panels shown in Figure 5B had q = 0.048.

The distributions of these six scores showed group-level shifts but retained substantial within-group overlap (Figure 5B). When the same marker panels were evaluated in GSE190023 and GSE238053, their directions varied, and no panel reached cohort-wise FDR significance in either validation cohort (Figure 5C). The sample-level heatmap showed local expression–score patterns rather than a binary case–control partition (Figure 5D). Because the scores were averages of marker-gene z values, they could not be translated into percentages or absolute numbers of immune or stromal cells.

Several RCD genes correlated with individual marker scores in the discovery cohort after the overlapping marker genes were removed (Figure 5E). These associations remained exploratory coexpression relationships and did not establish cell–cell interaction or causal coupling. Figure 5F showed that the discovered differentially expressed genes overlapped with terms related to cytokine response, cytokine production, inflammatory response, and cytokine-mediated signaling. The panel displays overlap counts only and was not interpreted as a separate formal enrichment test.

### 3.6 The three-gene classifier showed exploratory separation without perfect training performance

Five-fold stratified cross-validation selected C = 0.193 for the L1-penalized model, with a mean validation-fold AUC of 0.806 (Figure 6A). The coefficient paths showed that COL11A1, SPON2, and PITPNC1 moved away from zero as regularization was relaxed and were retained in the final three-gene combination (Figure 6B). The separate random-forest ranking placed UBXN11, EVA1A, SPON2, CCL8, AC092535, COL11A1, SSTR2, AK5, HTRA3, and CACNA1C among the 10 highest-importance features (Figure 6C); this ranking was descriptive and was not treated as an independent confirmation of the LASSO selection.

To remove the earlier disagreement between Figures 6D, 7, the revised cross-cohort display uses the same unique-symbol DESeq2 log2 fold changes in both figures. In GSE140731, GSE190023, and GSE238053, respectively, COL11A1 effects were +4.1, +2.1, and −1.1; SPON2 effects were +2.2, +1.8, and +1.9; and PITPNC1 effects were +1.3, +0.8, and +1.2 (Figure 6D). SPON2 and PITPNC1, therefore, retained positive directions across the three cohorts, whereas COL11A1 reversed direction in GSE238053.

Using pooled out-of-fold predictions, the GSE140731 AUC was 0.820 (95% CI, 0.684–0.930), replacing the earlier apparent training AUC of 1.000. In the small external cohorts, the AUC was 0.781 (95% CI, 0.444–1.000) for all AC versus control in GSE190023, 0.812 (95% CI, 0.400–1.000) for severe AC versus control in GSE190023, and 0.750 (95% CI, 0.250–1.000) for frozen shoulder versus control in GSE238053 (Figure 6E). The very wide confidence intervals reflected the limited number of samples and included values compatible with weak discrimination.

The discovery score distributions showed the clearest case–control separation, whereas the validation cohorts showed directional separation with marked dispersion (Figure 6F). Because feature ranking preceded the reported cross-validation and the discovery comparator differed strongly in age and sex, these results describe dataset separation only and do not establish clinical diagnostic accuracy.

### 3.7 Network structure and gene-wide replication were heterogeneous

After the removal of self-loops and reciprocal duplicates, the STRING network contained 30 nodes and 330 unique undirected edges (Figure 8A). IL1B and IL6 had the greatest node strength, followed by TP53, TLR4, JUN, CASP8, STAT3, TRAF6, NFKBIA, MAPK3, IL18, and IKBKB (Figure 8B). Many hub–RCD gene pairs were positively correlated in the discovery cohort, but negative associations were also present, including correlations involving JAK3 and MAPK12 targets (Figure 8C). Self-correlations were excluded before multiple-testing correction.

**FIGURE 8:**
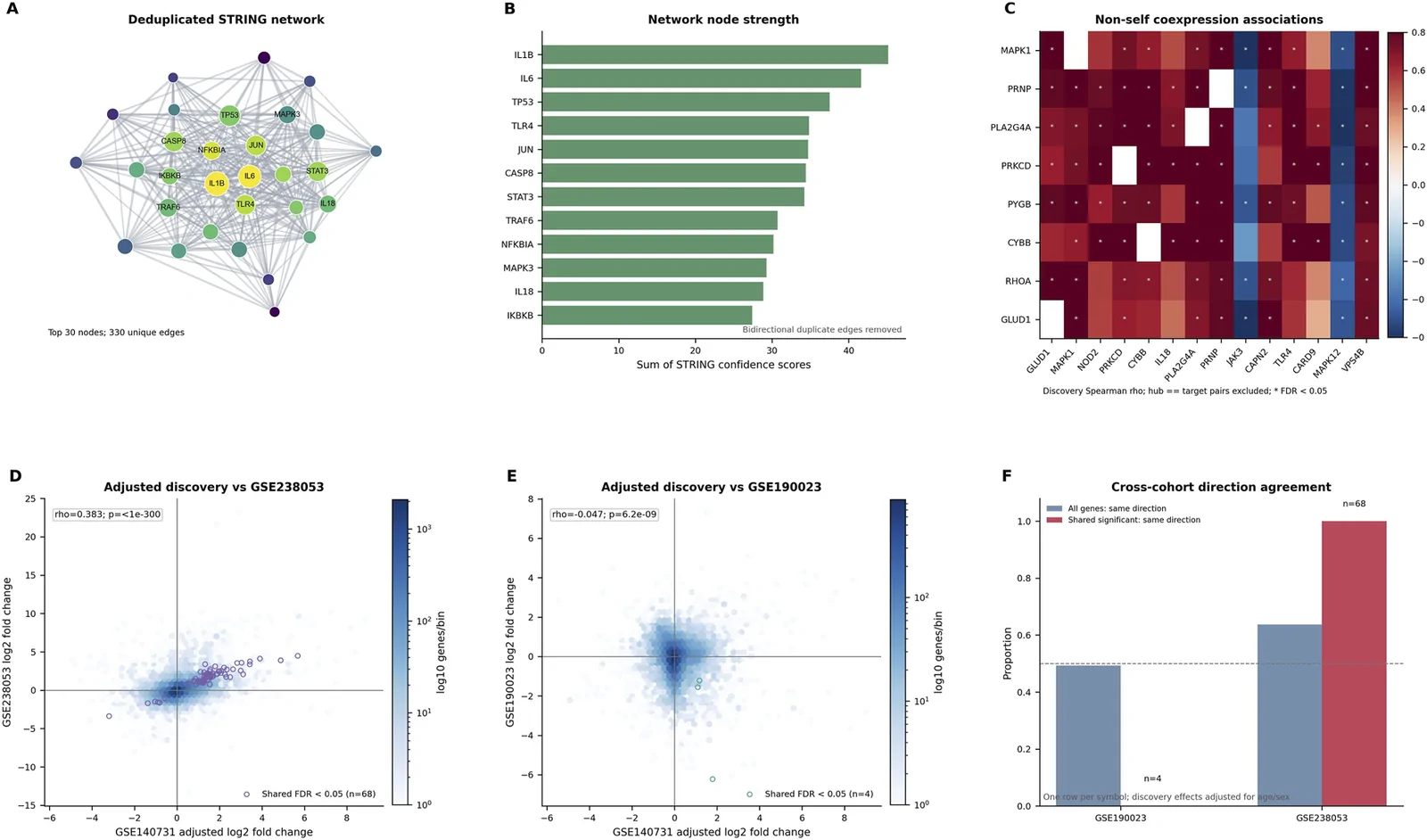
Deduplicated STRING network and cross-cohort gene-effect concordance. **(A)** STRING v11.5 network after removal of self-loops and reciprocal duplicate edges; the graph contains 30 nodes and 330 unique undirected edges. **(B)** Node strength, calculated as the sum of incident STRING combined scores. **(C)** Non-self Spearman correlations between network hubs and RCD-related genes in GSE140731; asterisks indicate FDR q < 0.05. **(D)** Density scatter plot comparing age/sex-adjusted GSE140731 log2 fold changes with GSE238053 effects (rho = 0.383; 68 genes significant in both cohorts). **(E)** Corresponding comparison with GSE190023 all-AC effects (rho = −0.047; four genes significant in both cohorts). **(F)** Proportion of matched genes with the same effect direction; values were 0.637 for GSE238053 and 0.493 for GSE190023. These panels describe cohort concordance and do not identify the source of discordance.

Gene-wide effects in adjusted GSE140731 showed a modest positive correlation with GSE238053 (Spearman rho = 0.383), and 68 genes were significant at FDR < 0.05 in both cohorts (Figure 8D). In contrast, adjusted discovery effects were essentially uncorrelated with GSE190023 all-AC effects (rho = −0.047), with only four genes significant in both datasets (Figure 8E). Same-direction proportions across all matched genes were 0.637 for GSE238053 and 0.493 for GSE190023 (Figure 8F). The near-zero GSE190023 correlation indicated lack of replication in that small cohort; it did not by itself identify a technical bias or prove that either cohort was erroneous.

### 3.8 Cross-cohort synthesis identified selected gene-level signals but no unified RCD program

The integrated selected-gene heatmap further illustrated both replication and heterogeneity (Figure 7A). In GSE140731, GSE190023, and GSE238053, respectively, the log2 fold changes were +4.8, +2.6, and +2.2 for POSTN; +4.1, +2.1, and −1.1 for COL11A1; +2.2, +1.8, and +1.9 for SPON2; +1.3, +0.8, and +1.2 for PITPNC1; +8.3, +7.0, and +5.0 for MMP9; +1.7, −1.8, and +2.2 for MAPK12; −0.9, −0.4, and −1.6 for PLA2G4A; +0.2, +0.2, and +3.3 for CCL21; and +0.6, −0.2, and +1.6 for SNAI1. MMP9 showed the most stable large positive effect, while COL11A1 and MAPK12 changed direction in one cohort.

Only two RCD-related genes were significant at FDR < 0.05 with the same direction in two cohorts: PLA2G4A was downregulated and MAPK12 was upregulated in both adjusted GSE140731 and GSE238053 (Figure 7B). The forest display confirmed consistently positive effects for MMP9; generally positive effects for SPON2, PITPNC1, and POSTN; and a negative GSE238053 effect for COL11A1, while also showing the broad confidence intervals in small cohorts (Figure 7C).

RCD score confidence intervals frequently crossed zero in the validation cohorts (Figure 7D). Direction-normalized AUCs were 0.53, 0.81, and 0.94 for pyroptosis; 0.72, 0.62, and 0.75 for ferroptosis; and 0.80, 0.75, and 0.56 for necroptosis in GSE140731, GSE190023, and GSE238053, respectively (Figure 7E). Because each value was defined as max (AUC, 1 - AUC), these numbers indicate only the magnitude of group separation after *post hoc* direction choice. They do not contradict the negative ferroptosis/necroptosis point estimates and do not demonstrate pathway activation or diagnostic utility.

The integrated concordance plot summarized the same cohort dependence: GSE190023 had rho = −0.047, 49.3% same-direction genes, and four shared significant genes, whereas GSE238053 had rho = 0.383, 63.7% same-direction genes, and 68 shared significant genes (Figure 7F). Together with the adjusted enrichment results, these findings support ECM-associated remodeling as the strongest biological signal in the present analysis, but they do not show that a single ECM pathway or RCD program reached formal significance in every cohort.

### 3.9 qPCR provided partial, model-specific support for selected candidates

In the TGF-β1-stimulated SW982 model, the mean relative COL11A1 expression was 1.888 under the profibrotic condition and 1.036 in vehicle-control cells (P = 0.0006). SPON2 expression was also higher after TGF-β1 exposure (1.518 versus 1.016; P = 0.0040). PITPNC1 showed a smaller numerical increase (1.240 versus 1.010) that was not statistically significant (P = 0.272). PLA2G4A expression was lower after stimulation (0.700 versus 1.013; P = 0.0065; Figure 9).

**FIGURE 9:**
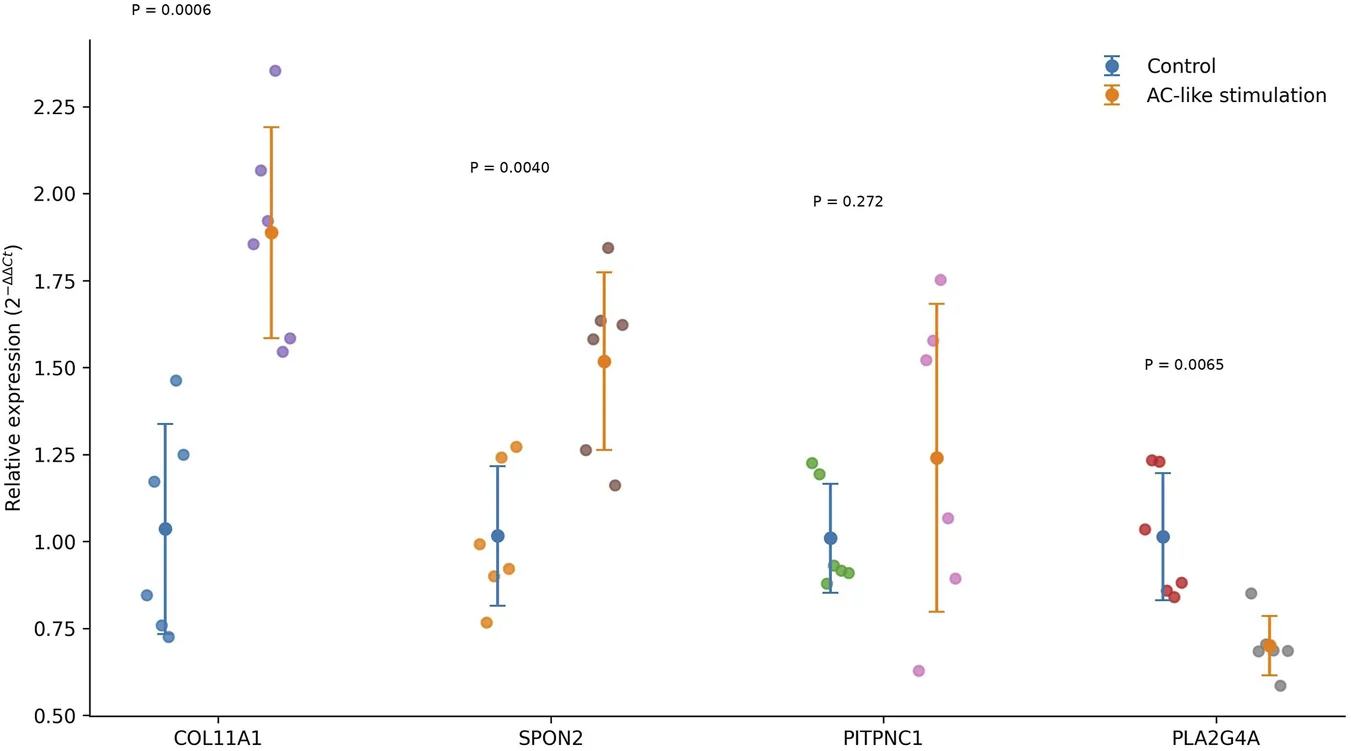
qPCR validation in a TGF-β1-stimulated SW982 cell-line model. Relative expression of COL11A1, SPON2, PITPNC1, and PLA2G4A is shown from the left to the right for vehicle-control and TGF-β1-induced profibrotic conditions. Six independent culture replicates per condition, generated across three separate experimental runs with two independently seeded wells per condition in each run, are displayed on the 2^−ΔΔCT^ scale. Each cDNA sample was analyzed in technical triplicate, and technical Ct values were averaged before relative-expression calculation. Points represent independent culture replicates, and summary bars/markers show mean ± SD. P-values are from two-sided Welch t-tests. Mean stimulated-versus-control values were 1.888 versus 1.036 for COL11A1 (P = 0.0006), 1.518 versus 1.016 for SPON2 (P = 0.0040), 1.240 versus 1.010 for PITPNC1 (P = 0.272), and 0.700 versus 1.013 for PLA2G4A (P = 0.0065). The experiment supports COL11A1 and SPON2 upregulation and PLA2G4A downregulation in this model; PITPNC1 was not significantly different.

Thus, the cell-line experiment supported the directions of COL11A1, SPON2, and PLA2G4A in this TGF-β1-induced profibrotic model, but it did not reproduce PITPNC1. The non-significant PITPNC1 result is not a mathematical contradiction of multivariable feature selection because the classifier used the joint information from three genes rather than requiring each gene to be significant in a separate cell experiment. It nevertheless limits the claim of biological validation: the three-gene panel should be described as computationally selected, with only two classifier genes supported by the current qPCR experiment.

## 4 Discussion

This three-cohort transcriptomic reanalysis shifts the interpretation of AC away from a uniform RCD model and toward an ECM-centered framework. The principal findings were: (i) age/sex-adjusted discovery differential expression was dominated by matrix-associated genes; (ii) ECM–receptor interaction remained strongly enriched after demographic adjustment; (iii) pyroptosis, ferroptosis, and necroptosis summaries showed different directions and levels of significance rather than coordinated activation; (iv) immune marker–gene associations were evident in the discovery cohort but did not reproduce at cohort-wise FDR significance in the small validation cohorts; (v) gene-level cross-cohort concordance was heterogeneous; and (vi) the three-gene classifier separated groups in the analyzed datasets but remains exploratory because of small validation samples and non-nested feature selection.

ECM remodeling was the most internally coherent biological signal. In the adjusted GSE140731 ranking, ECM–receptor interaction was positively enriched, and the upregulated discovery annotations were dominated by extracellular-structure and collagen-related terms. This observation is consistent with established frozen-shoulder biology. Patient-derived capsule studies have demonstrated activated stromal fibroblasts and inflammatory cytokine dysregulation (Akbar et al., 2019), while primary synovial fibroblasts from frozen shoulder show increased ECM production and an IL-6/PI3K-Akt-linked fibrotic phenotype (Yang R. et al., 2022). Single-cell analysis further identifies macrophage–fibroblast interactions associated with matrix remodeling and fibrosis resolution (Ng et al., 2024). Earlier pathology and gene-expression studies likewise documented myofibroblast-rich fibrosis, collagen deposition, growth-factor/MMP dysregulation, and increased matrix-associated transcripts in frozen-shoulder tissue (Bunker and Anthony, 1995; Rodeo et al., 1997; Bunker et al., 2000; Hand et al., 2007; Cohen et al., 2016; Nishimoto et al., 2022). Thus, the present cross-cohort results strengthen an ECM-centered interpretation without requiring a specific cell-death pathway to be uniformly activated.

The RCD results require cautious interpretation. The adjusted discovered pyroptosis score was not different between groups, ferroptosis was lower with borderline FDR significance, and necroptosis was lower; the adjusted core necroptosis gene set was likewise negatively enriched. These transcriptomic summaries do not demonstrate cell-death execution. Mechanistically, RCD could be either upstream of inflammation or secondary to an already inflammatory and oxidatively stressed tissue environment. In rheumatoid arthritis, synovial NLRP3/caspase-1/GSDMD activity and gasdermin-dependent pyroptosis have been linked experimentally to inflammatory disease activity (Zhang et al., 2023; Zhai et al., 2022). In inflammatory arthritis and osteoarthritis-related models, ferroptotic phenotypes can be modified by pathway-directed interventions; GPX4-associated mechanisms are implicated, and ferrostatin-1 rescue has been demonstrated in temporomandibular-joint osteoarthritis models (Chang et al., 2022; Liu et al., 2024; Chen et al., 2024). Necroptotic DAMP release also provides a plausible route from membrane injury to inflammation and fibrosis (Liu et al., 2022; Yang J. et al., 2022). These studies establish biological plausibility in analogous musculoskeletal settings, but they cannot be taken as evidence that the same causal sequence occurs in AC.

The immune analyses should also be read within their measurement limits. The scores used here are global-z summaries of curated marker genes, not deconvolution-derived estimates of immune-cell fractions. Although several marker sets, including NK-cell, endothelial-cell, and monocyte signatures, were associated with the case status in the adjusted discovery analysis, none reached cohort-wise FDR significance in the validation datasets. This pattern is compatible with genuine context-dependent immune remodeling, but it may also reflect differences in tissue sampling, disease severity, cellular composition, and small sample sizes. Tissue studies showing cytokine elevation, MAPK/NF-kappaB activation, and inflammatory-cell infiltration further support the presence of local inflammatory signaling in AC, while not establishing uniform immune-cell abundance changes (Rodeo et al., 1997; Hand et al., 2007; Kanbe et al., 2009; Kabbabe et al., 2010; Lho et al., 2013). The single-cell atlas of frozen shoulder demonstrates that macrophage states and fibroblast subtypes are heterogeneous within the diseased capsule (Ng et al., 2024), underscoring why bulk marker scores should not be interpreted as direct cell-abundance measurements.

Cross-cohort heterogeneity is a central result rather than a technical inconvenience. GSE140731 uses shoulder-instability surgical controls rather than healthy controls and shows pronounced age and sex imbalance; the primary discovery model, therefore, adjusted for both covariates. The two validation cohorts are small and differ in tissue source and clinical grouping. Consistent with these design differences, adjusted discovered effects correlated only modestly with GSE238053 and essentially not at all with GSE190023. These observations argue against a single, uniformly replicated transcriptomic program and favor conclusions that explicitly depend on cohort context.

The three-gene COL11A1–SPON2–PITPNC1 model should not be interpreted as a clinical diagnostic test. Using pooled out-of-fold predictions, the discovery AUC was 0.820 (95% CI, 0.684–0.930), while external-cohort AUCs ranged from 0.750 to 0.812 and had very wide confidence intervals because of the small samples. In addition, candidate-pool preselection preceded the reported cross-validation workflow, so the validation framework was not fully nested. The model, therefore, provides an exploratory summary of separability in these datasets rather than evidence of clinical utility. Prospective evaluation in larger, independently recruited populations with prespecified features, fully nested model development, calibration assessment, and comparison against routine clinical variables would be required before diagnostic claims could be considered.

Figure 9 adds a model-specific experimental layer to the transcriptomic analyses. In the SW982 qPCR experiment, COL11A1 and SPON2 were increased and PLA2G4A was decreased after TGF-β1 stimulation in the profibrotic model, whereas PITPNC1 was not significantly changed. The non-significant PITPNC1 result also illustrates that computational selection does not guarantee experimental replication. These findings provide experimental support for selected gene-expression changes in this cell-line model, but they should not be interpreted as proof of RCD pathway execution, causal relevance to AC, or clinical utility.

Several limitations define the boundary of inference. First, the discovery comparator is a younger, more male shoulder-instability surgical population rather than a healthy control group; age/sex adjustment reduces but cannot eliminate confounding. Second, both validation cohorts are small, which limits power and makes effect estimates, enrichment results, and AUCs unstable. Third, bulk RNA-seq cannot resolve whether the observed expression differences reflect transcriptional regulation within cells or shifts in cellular composition. Fourth, RCD gene-set scores and individual transcripts are not direct measurements of pyroptotic pore formation, lipid-peroxidation-dependent ferroptosis, or RIPK3/MLKL-mediated necroptotic execution. Fifth, the immune scores are marker–gene summaries rather than cell fractions. Finally, the study is observational and does not establish causal directionality. Future work should combine independent clinical cohorts with spatial or histologic validation and functional experiments in primary AC fibroblasts and macrophages using pathway-specific perturbation and rescue assays. Until such evidence is available, the RCD and immune findings should be regarded as hypothesis-generating associations, whereas ECM remodeling represents the most reproducible signal in the present data. Figure 9 is limited to a single SW982 cell-line experiment and requires independent validation in primary AC cells or tissues.

## 5 Conclusion

Across three independent bulk RNA-seq cohorts, extracellular-matrix remodeling emerged as the most consistent transcriptional feature of adhesive capsulitis. Pyroptosis-, ferroptosis-, necroptosis-, and immune-related signatures varied by cohort and, in several analyses, showed null or negative rather than uniformly positive effects. These findings do not establish execution or causal crosstalk of RCD pathways in AC. The COL11A1–SPON2–PITPNC1 classifier is exploratory and should not be considered a clinical diagnostic model. Overall, the data support an ECM-centered, cohort-aware interpretation of AC and identify RCD and immune associations as priorities for independent functional validation rather than established therapeutic or prognostic targets. Figure 9 provides model-specific qPCR validation of selected genes, but broader functional and tissue-level validation remains necessary; the principal disease-level conclusions continue to rest on the three public transcriptomic cohorts.

## Data Availability

The public transcriptomic datasets analyzed in this study are available in the Gene Expression Omnibus (GEO) under accession numbers GSE140731, GSE190023, and GSE238053. The raw qPCR data generated in this study are available from the corresponding authors upon reasonable request.
